# A systematic analysis of neurons with large somatosensory receptive fields covering multiple body regions in the secondary somatosensory area of macaque monkeys

**DOI:** 10.1152/jn.00241.2016

**Published:** 2016-08-24

**Authors:** M. Taoka, T. Toda, S. Hihara, M. Tanaka, A. Iriki, Y. Iwamura

**Affiliations:** ^1^Department of Physiology, Toho University School of Medicine, Tokyo, Japan;; ^2^Section of Cognitive Neurobiology, Department of Maxillofacial Biology, Tokyo Medical and Dental University, Tokyo, Japan; and; ^3^Laboratory for Symbolic Cognitive Development, RIKEN Brain Science Institute, Wako, Saitama, Japan

**Keywords:** awake macaque monkey, secondary somatosensory area, large receptive field, single-unit recording

## Abstract

Previous neurophysiological studies performed in macaque monkeys have revealed complex somatosensory responses in the secondary somatosensory area (SII), such as large receptive fields (RFs), as well as bilateral ones. However, systematic analyses of neurons with large RFs have not been performed. In the present study, we recorded single-unit activities in SII of awake macaque monkeys to investigate systematically large RFs by dividing the whole body into four body regions (head, trunk, forelimb, and hindlimb). Recorded neurons were classified into two types, according to whether the RFs were confined to one body region: single (*n* = 817) and combined (*n* = 282) body-region types. These two types were distinct in terms of the percentage of bilateral RFs: 55% in the single-region type and 90% in the combined type, demonstrating that two types of RF enlargement occur simultaneously in the combined type, namely, RF convergence from different body regions and RF convergence from both hemibodies. Among the combined-type RFs, two tendencies of RF convergence were found: *1*) the distal parts of the limbs (i.e., hand and foot) and the mouth are interconnected, and *2*) the trunk RFs extend continuously toward the distal parts of the limb and head to cover the entire body surface. Our distribution analysis on unfolded maps clarified that neurons having RFs with these two tendencies were distributed within specific subregions in SII.

## Abstract (precis)

*Receptive fields (RFs) of the secondary somatosensory cortex of Japanese monkeys were analyzed. We found large RFs, mostly bilateral ones, covering more than one body region when the entire body was divided into the four: forelimb, hindlimb, trunk, and head. Two tendencies of RF enlargement—interconnecting limb extremities and the mouth and expansion of the trunk RF toward limb extremities to cover the entire body—were found. Neurons with either tendency were distributed in a specific subregion*.

## NEW & NOTEWORTHY

*Receptive fields (RFs) of the secondary somatosensory cortex of Japanese monkeys were analyzed. We found large RFs, mostly bilateral ones, covering more than one body region when the entire body was divided into the four: forelimb, hindlimb, trunk, and head. Two tendencies of RF enlargement—interconnecting limb extremities and the mouth and expansion of the trunk RF toward limb extremities to cover the entire body—were found. Neurons with either tendency were distributed in a specific subregion*.

it is often said that neurons in the secondary somatosensory area (SII), located in the upper bank of the lateral sulcus (LS), show more complex response properties to somatic stimulation than the primary somatosensory cortex [SI; e.g., stimulus selectivity, attentional effects on neural activity, bilateral integration from both hemibodies, and large receptive fields (RFs) covering multiple body parts]. Single-unit recording studies using awake macaque monkeys have been undertaken to elucidate response selectivity to tactile stimuli (Fitzgerald et al. 2004, 2006a, b; Hsiao et al. 2002; Jiang et al. 1997), effects of attention on neural activity (Burton et al. 1997; Hsiao et al. 1993; Meftah et al. 2002, 2009; Poranen and Hyvarinen 1982; Roy et al. 2007), and bilateral RFs encompassing the body midline (Robinson and Burton 1980a, b; Whitsel et al. 1969). These studies showed that SII neurons have more complex response properties than SI neurons, supporting the idea that SII is engaged in more complex information processing than SI (Hsiao 2008; Iwamura 2000; Iwamura et al. 2001a). As for SII neurons with large RFs, however, no systematic studies have been done; only anecdotal descriptions of RF configurations have been reported, such as RFs covering the trunk and adjacent portions of limbs (Krubitzer et al. 1995; Poranen and Hyvarinen 1982; Robinson and Burton 1980a, b; Whitsel et al. 1969).

Hierarchical information processing has been studied intensively in the postcentral somatosensory cortex. In a series of studies by Iwamura and colleagues using awake macaques, it was revealed that response complexity, such as RF size and the stimulus selectivity of neurons, increases along the rostrocaudal axis in the postcentral somatosensory area (Iwamura 1998; Iwamura et al 2001a, 2002). To evaluate quantitatively RF convergence in the area representing the hand, Iwamura et al. (1993) divided the distal part of the forelimb into several areas (e.g., digits I–V, ulnar palm, radial palm) and counted the number of areas of the hand covered by the RFs. In more medial regions representing the proximal forelimb, trunk, and hindlimb, Taoka et al. (1998, 2000) performed a similar analysis of RF size. They divided the body parts into several areas—the forelimb was divided into the hand, forearm, and upper arm, and the hindlimb was divided into the foot, leg, and thigh—to evaluate quantitatively RF convergence. These studies revealed that RF convergence from different regions of the body progresses toward the caudal end of the postcentral gyrus and terminates in areas 2 and 5; multidigit RFs, large RFs covering more than one body region (e.g., the trunk and limbs, the forelimb and hindlimb), as well as bilateral RFs appeared throughout the caudal end of the postcentral gyrus.

The complex RF responses to tactile stimulation have been studied in the SII area representing the hand and showed texture selectivity (Chapman et al. 2002; Jiang et al. 1997) and directional preference (Fitzgerald et al. 2004, 2006a, b; Hsiao et al. 2002). Hsiao and colleagues (2002) conducted a number of experiments. Hsiao (2008) reviewed their works and concluded that SII hand neurons process size and shape information and therefore, contribute to tactile-object recognition. Such elaborate experiments have explored only hand representation; thus the response properties of large RFs covering multiple body areas remain to be clarified. Because RF convergence that generates large RFs has been shown in both the postcentral somatosensory area and SII, the question arises as to whether there are any differences between these two somatosensory areas in their RF convergence characteristics.

The present study was performed to resolve this question. We divided the entire body into several regions to allow a systematic analysis of the extent of RF convergence. Additionally, we investigated the distribution of neurons with large RFs on an unfolded map of SII. We identified two novel forms of RF convergence, neither of which had been found in the postcentral somatosensory cortex. In addition, neurons exhibiting these RF convergence features were distributed in specific subregions in SII.

## METHODS

### 

#### Subjects and surgery.

The results reported here are based on chronic experiments performed in nine hemispheres of six male Japanese monkeys (*Macaca fuscata*). All experimental procedures were approved by the Animal Care and Use Committees of Toho University School of Medicine, Tokyo Medical and Dental University, and RIKEN, and they were also in accordance with the NIH *Guide for the Care and Use of Laboratory Animals*.

Before surgery, the monkeys were trained to accept natural stimulation to each body part without struggling. Surgery was performed in two steps under deep anesthesia with pentobarbital sodium (30 mg/kg). The initial step was to implant devices on the skull to fix the monkey's head to the monkey chair. Approximately 1–2 wk later, a trephine opening of ∼2 cm in diameter was made in the skull over the most lateral part of the postcentral gyrus. A cylindrical chamber (20 mm in diameter) was placed over the opening perpendicular to the skull surface and fixed to the bone with small screws and dental acrylic. The chamber was filled with saline and closed with a plastic cap.

#### Neural recordings and identification of somatosensory RFs.

Single neuron activity was recorded through glass-coated, platinum-iridium electrodes or commercially obtained tungsten microelectrodes (FHC, Bowdoin, MA) with resistance of 3–8 MΩ. At the beginning of each recording session, the base plate for a microdrive, which had *x-y*^**^ scales of 100 μm steps (MO-95; Narishige, Tokyo, Japan), was attached to the cylinder on the skull. Penetrations were made almost perpendicularly to the cortical surface and spaced at ∼1 mm intervals. One to three electrolytic lesions were made at the ends of selected penetrations to facilitate later histological identification and reconstruction of the penetrations.

The monkey chair used in the present study was specially designed for RF investigation of large areas of the body. The monkey sat on two parallel stainless-steel cylinders (15 cm long, 4 cm in diameter), positioned ∼4 cm apart, while the lower body was restrained around the waist with stainless-steel pipes (2 cm in diameter). To restrain the movements of the trunk, the monkey's neck passed through a hole made with two horizontal acrylic plates, one of which could be moved back and forth to adjust the size of the hole. These apparatuses, as well as the head-fixing device, were supported by a vertical, metallic pole (4 cm in diameter). This monkey chair enabled the experimenter to access almost the entire body surface and to manipulate joints, even the hip and shoulders, in various directions.

RFs and submodality preferences of neurons were examined using a variety of natural stimuli, such as light touching, stroking, tapping, kneading, or pressing, applied with the experimenter's fingers, a hand-held metallic probe, or other tools. We manipulated many joints when the monkeys were relaxed. To confirm whether a neuron responded to the joint movements, we carefully checked that touch or deformation of the skin around the joint did not activate the neuron. In the case of the hip joint, such determination was more difficult than for other joints, because manipulation of the hip joint could simultaneously stimulate the monkey's skin surface attached to the chair. Nevertheless, we judged that a neuron certainly responded to the manipulation of the hip joint if stimuli applied to the skin surface attached to the chair were not effective. We also tried to avoid twisting the trunk during the manipulation of the hip joint so that the extent of the rotation of the hip joint was limited. Thus the number of neurons responding to the manipulation of the hip joint might have been underestimated in our samples compared with the number of neurons responding to the manipulations of other joints.

**Fig. 1.**
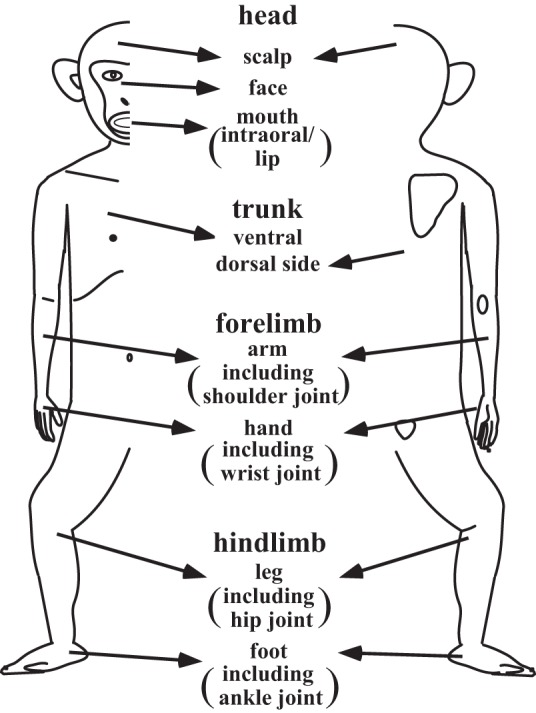
Definitions of the body regions used for the identification of receptive fields. The entire body is divided into 4 regions: head, trunk, forelimb, and hindlimb. Each region is further subdivided into 2 or 3 subregions. The arrows indicate the location of each subregion. The schematic drawing of the hemibody on the *left* shows the ventral side and that on the *right* shows the dorsal side.

We defined four body regions—head, trunk, forelimb, and hindlimb—and each region was further divided into two or three subregions (Fig. 1). RF extents were examined as much as possible, but a part of the sample was not determined, precisely because of the following three limitations: *1*) difficulty in accessing the body area (e.g., deep intraoral structures)—we trained animals to accept stimuli applied to the intraoral structures, but we had to forgo stimulation of the deepest intraoral areas to avoid animals' struggles against stimuli; *2*) unstable responsiveness to repeated stimuli, as reported in earlier studies (Robinson and Burton 1980a; Taoka et al. 2013; Whitsel et al. 1969)—to ensure constant neural responses, we applied repeated stimuli with sufficient interstimulus intervals (2–3 s) to overcome this limitation, but the determination of RFs under such conditions was time consuming and difficult; *3*) complexity of stimuli needed to activate neurons—for example, we often observed that pressing the skin surface of the hand or foot was effective only when the animal's hand or foot was placed on the horizontal pipe of the chair. Thus in some cases, the RF extent could not be determined precisely because of any one of these three limitations. In such cases, if the neuron's RF was confined, at least within a certain subregion of the body shown in Fig. 1, then we classified it as an RF in that RF subregion.

We divided the recorded neurons into different groups, according to the differences in RF properties, such as the number of body regions covered by RFs and the laterality of RFs (see results). The χ^2^-test was applied to evaluate the statistical difference between the groups with respect to a certain characteristic, such as the difference in the number of neurons with bilateral RFs.

#### Estimation of recording sites and analyses of distributions on unfolded maps.

After one or both hemispheres were explored for ∼2–4 mo, the animal was killed with an overdose of pentobarbital sodium and perfused transcardially with 0.9% saline, followed by 10% formalin. Before the brain was removed, three guide wires were inserted into the brain through the microdrive attached to the cylinder to indicate the orientation of penetrations. We also used marks on the guide wires on the cortical surface as reference points to identify each electrode penetration. After dehydration and celloidin embedding, 40 μm-thick frontal sections were made for six of the nine hemispheres. The remaining three hemispheres were sectioned at a right angle to the LS. Every section was stained for Nissl substance, and we searched for gliosis around the electrode tracks and electrolytic lesions. Because most electrode trajectories traversed many sections, they were traced by superimposing serial sections. Each penetration was assigned to one of the electrode tracks by guidance from its surface location. The ratio of brain shrinkage associated with the histological procedures was estimated by the overall shrinkage of the brain block. The depth of the recording site of each unit was estimated based on the distance reading of the electrode manipulator during the experiments and the ratio of brain shrinkage.

The borders of SII with the surrounding cortical areas (e.g., the oral region of SI and area 7b) were determined histologically and physiologically. In this study, we refer to SII as the classically defined somatosensory area in the upper bank of the LS that includes both the parietal ventral area and SII, as demonstrated to exist in macaque monkeys by Krubitzer et al. (1995). The cytoarchitectural identification based on Nissl-stained sections followed the criteria described by Burton et al. (1995), Friedman et al. (1980), and Jones and Burton (1976). Procedural details of the areal discriminations are described elsewhere (Hihara et al. 2015; Taoka et al. 2013). The border between the SI oral representation and SII was not difficult to delineate, but it was difficult to draw clear borders with area 7b, because the neurons of SII and those of area 7b intermingle at their borders (Hihara et al. 2015). According to the somatotopic organization in SII described in previous studies (Burton et al. 1995; Fitzgerald et al. 2004, 2006a, b; Hihara et al. 2015; Krubitzer et al. 1995; Robinson and Burton 1980a; Taoka et al. 2013), the caudal region of SII near the boundary of area 7b represents the proximal forelimb, trunk, and hindlimb. Therefore, the region whose neurons almost always represented those body parts was considered to be in SII. When additional caudal regions were explored, and other representations (e.g., the face or hand) appeared, we determined that the electrode might have entered the bordering region with area 7b, where the neurons of SII and area 7b are probably intermingled, so we excluded the neurons recorded there from the present study sample. As for the rostral part of SII, we only explored the region caudal to the lateral tip of the central sulcus to ensure that only SII neurons were included (Taoka et al. 2013). Accordingly, the regions explored in the present study did not completely cover both ends of the caudal- and rostral-most areas of SII.

**Fig. 2.**
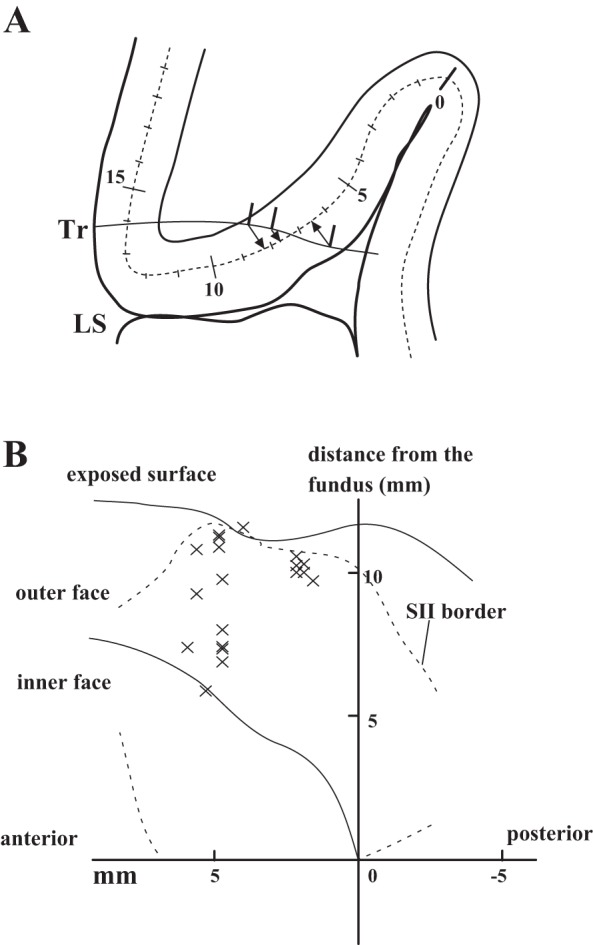
Reconstruction of recording sites on a 2-dimensional, unfolded map. Distributions of recorded neurons were analyzed on 2-dimensional, unfolded maps. *A*: estimated recording sites (thick vertical lines along the electrode track) were plotted on traces of histological sections where the line of layer IV was drawn (dashed line). The recording sites were projected onto the layer IV line (arrows), and the distances from the lateral sulcus (LS) upper fundus (the border between the insular cortex and the upper bank of the LS) were measured along the layer IV line and used as *y* values. The location along the rostrocaudal axis of the histological section was defined as the *x* value when the caudal end of the insular cortex was 0. Numerals along the layer IV line indicate the distance (millimeters) from the LS upper fundus. Tr, electrode track. *B*: recording sites were plotted on the unfolded map based on their *x-y* values. “Hand & head” neurons of the *limb type* recorded in 1 hemisphere were plotted as examples. The *x*-axis and *y*-axis correspond to the rostrocaudal axis (*left* direction is the anterior direction) and the distance from the upper fundus of the LS along layer IV, respectively. The border of the secondary somatosensory area (SII) was drawn with a dashed line (SII border). Because of the difficulty in delineating a clear border, the areal border could not be drawn in the caudal- and rostral-most ends of SII. The boundary between the exposed cortical surface and the upper bank of the LS and that between the inner and outer faces of the operculum are drawn with thin lines. Inner face, deeper part of the operculum facing the insular cortex; outer face, shallower part of the operculum facing the temporal lobe.

We analyzed the distributions of the recorded neurons on the two-dimensional maps, according to the method of Burton et al. (1995). After estimated recording sites were plotted on traces of histological sections, we drew the lines of layer IV (Fig. 2*A*). The recording sites were projected to the layer IV line, and the distances from the LS upper fundus (the border between the insular cortex and the upper bank of the LS) were measured and used as *y* values. The location along the rostrocaudal axis of the histological section was defined as the *x* value when the caudal end of the insular cortex was zero. Then, we constructed *x-y* coordinate maps of SII (Fig. 2*B*). After plotting data from different hemispheres on the same map, the center of gravity of the plotted neurons was calculated, the distance between the center and the recording site of every neuron was measured, and then an envelope was drawn to include 80% of neurons within it. For this distribution analysis, we only used data from the hemisphere where the frontal sections were made. The three hemispheres whose brain blocks were sectioned perpendicularly to the LS were excluded.

## RESULTS

### 

#### General descriptions of isolated neurons.

We isolated 1,975 neuronal units from nine hemispheres of six awake animals. Of these, the RFs and submodality preferences of 1,099 neurons were identified. Of these identified neurons, neurons with bilateral RFs (*n* = 704; 64%) were the most frequently observed. We also found 378 contralateral (34%) and 17 ipsilateral (2%) RF neurons. As for the submodality preferences, we found 578 skin neurons (53%), 488 deep neurons (44%), and 33 submodality convergence-type neurons (3%).

Among the 1,099 identified neurons, we often found neurons whose RF extents could not be determined precisely, because of the three limitations described in methods. For example, we often encountered neurons that responded only to complex stimuli to the hand or foot, such as those responding to pressure on the hand only when the monkey touched the horizontal stainless pipe of the chair and those responding when the experimenter forced the monkey's hand to slide along the pipe. We also detected neurons that responded when the fingers were forced open when the monkey grasped a certain object, such as the pipe of the chair. We classified these neurons as deep neurons with RFs on the hand or foot. We observed such not well-defined RF responses in 195 neurons. The percentages of skin and deep submodality neurons among these 195 neurons were quite different from those of the total sample: 18% skin (*n* = 36) and 77% deep (*n* = 150) neurons, compared with 53% and 44%, respectively, among the total neuron sample. The main focus of the present study was on RF laterality and RF positions. Therefore, we combined the well-defined and not well-defined RF neurons in the later analysis.

Among the remaining neurons whose RF and submodality preference could not be identified (*n* = 876), we found 210 neurons that were activated during active movements of the forelimb or hindlimb, such as when reaching to get a piece of food. Of these, 64% showed bilateral activity associated with limb movements, and 33% were active only during contralateral limb movements. Neurons with ipsilateral activity were rare (3%).

#### Comparisons of RF properties between single-region and combined types.

**Fig. 3.**
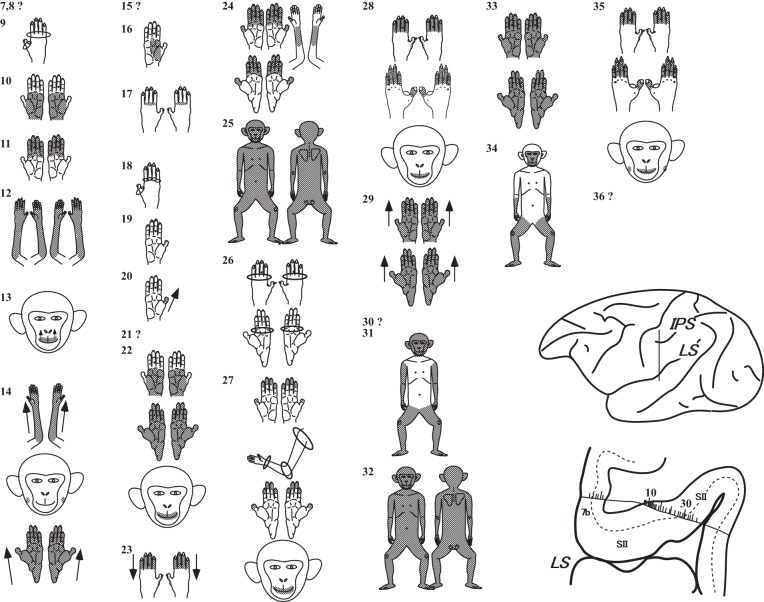
Examples of receptive fields (RFs) of neurons recorded from 1 penetration. Shaded areas indicate the extents of RFs. Effective positions of joint neurons are circled. Arrows indicate the preferred direction of moving stimuli on the skin surface. The estimated locations of 30 neurons are plotted and numbered (Nos. 7–36) along the electrode track, shown in the histological section. The location of the section is indicated by a vertical line in the lateral view of the hemisphere. Dotted lines indicate layer IV.?, RFs that could not be identified; LS, lateral sulcus; IPS, intraparietal sulcus; SII, secondary somatosensory area; 7b, area 7b [adapted from Iwamura (2001) and Iwamura et al. (2001b) with permission].

Figure 3 shows examples of the RFs of neurons recorded from an electrode penetration in one hemisphere. This electrode entered area 7b of the exposed cortical surface and then reached the deeper part of the operculum. Among 30 neurons isolated in SII, the RFs of 24 neurons could be determined.

RF positions and extents varied among neurons. All RFs, except No. 13, included the forelimb; 10 RFs were exclusive to the forelimb (Nos. 9–12, 16–20, and 23), and the remaining 13 RFs included other body regions, such as the head, hindlimb, and trunk. We found neurons with very large RFs covering almost the entire body surface (Nos. 25 and 32).

**Fig. 4.**
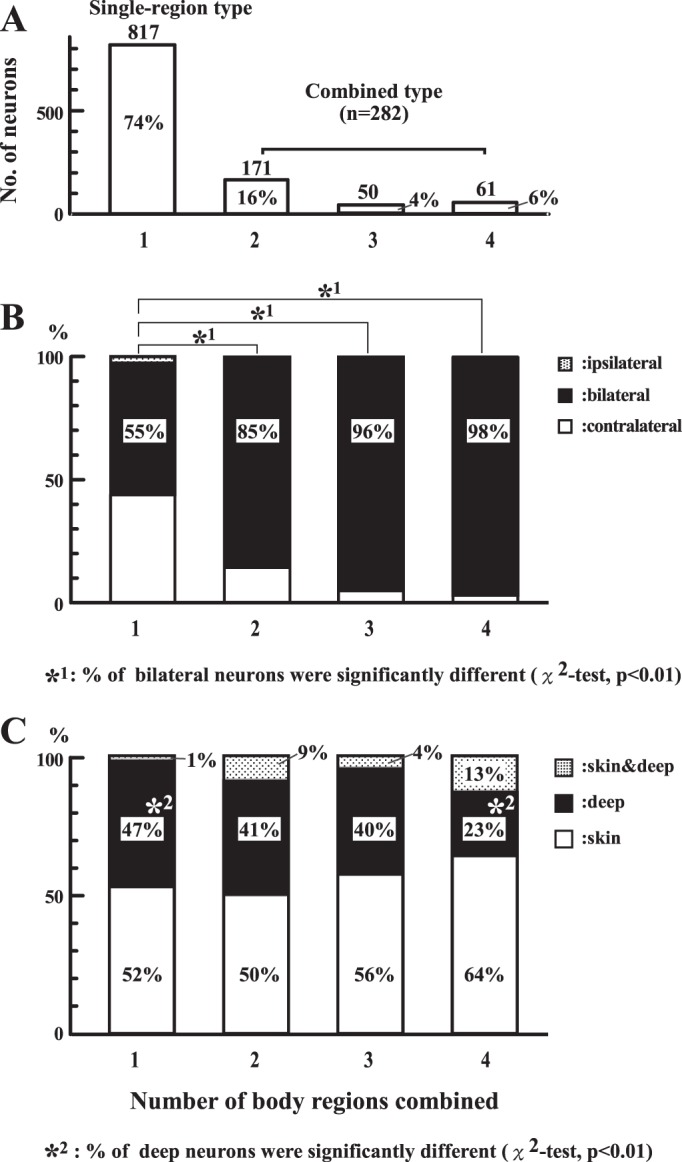
Comparison of neurons classified by the number of body regions covered by their receptive fields (RFs). Identified neurons were divided into 4 groups, according to the number of body regions that their RFs covered. The number of neurons in each group (*A*), comparisons of RF laterality (*B*), and submodality preferences (*C*) are shown. The 1,099 neurons are divided into 2 categories according to whether the RFs are confined to 1 body region (“single-region type”) or covered >1 body region (“combined type”; *A*).

First, we classified the identified neurons according to the number of body regions covered by their RFs when the body was divided into the four regions described in Fig. 1. For example, neurons with RFs restricted to one of the four body regions were classified as “one body-region” neurons (e.g., Nos. 9–13, 16–20, and 23), and those with RFs covering all four body regions were classified as “four body-region” neurons (e.g., Nos. 25 and 32). As shown in Fig. 4*A*, the most numerous neurons (*n* = 817; 74%) had RFs restricted to only 1 body region, followed by 171, 2 body-region neurons (16%); 50, 3 body-region neurons (4%); and 61, 4 body-region neurons (6%).

Among the RFs of the 817, 1 body-region neurons, 43% were contralateral, 55% were bilateral, and 2% were ipsilateral (Fig. 4*B*). The percentage of bilateral RFs increased as the number of body regions increased (85–98%), and no ipsilateral RF was found among multiple body-region neurons. χ^2^-Tests clarified that bilateral RFs were more commonly observed in every group of multiple body-region neurons than in one body-region neuron (*P* < 0.01). Thus we divided the 1,099 identified neurons into 2 groups (Fig. 4*A*): the single-region type (RF restricted to 1 body region, *n* = 817) and the combined type (RF covered >1 body region, *n* = 282). Neurons with bilateral RFs occupied the vast majority of the combined type (90%), indicating that two types of RF enlargements were observed in the combined type, that is, RF convergence from different body regions and RF convergence from both hemibodies.

Figure 4*C* shows the results of the analysis of submodality preferences in the four groups. The percentage of deep submodality neurons decreased as the number of body regions increased, but a significant difference was found only between the single-region (47%) and four body-region (23%) neurons. When statistical analysis was applied to the comparison of the single-region and combined types, deep neurons were found to be less common in the combined type (37%, χ^2^-test, *P* < 0.01). As for the submodality convergence type (“skin & deep” in Fig. 4*C*), the percentage of the single-region type (1%) was lower than any of the other three neuron groups, which resulted in the submodality convergence type being significantly more common in the combined type (9%) than in the single-region type (χ^2^-test, *P* < 0.01). We could not find any differences in the percentages of the skin submodality.

**Fig. 5.**
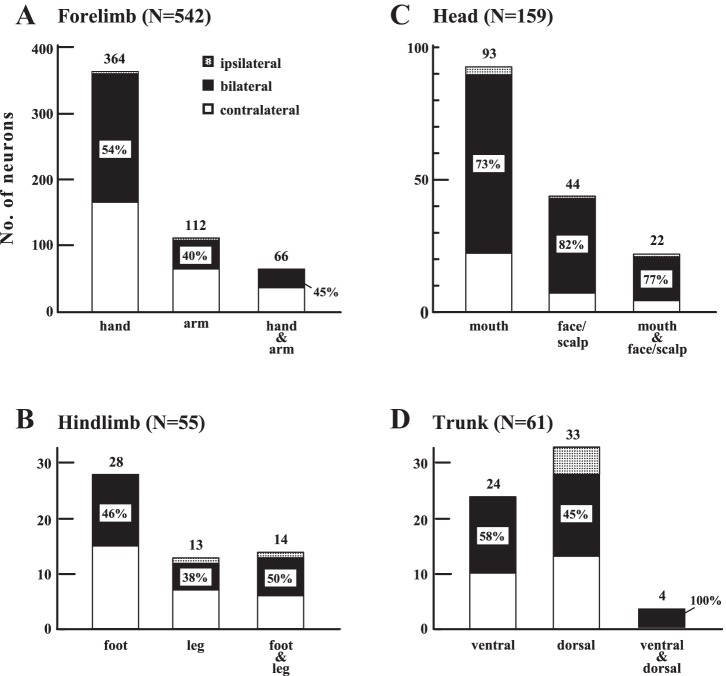
Comparison of receptive-field (RF) laterality between the convergence RF and single RF types among neurons of the single-region type. Neurons of the single-region type are classified into 4 groups: forelimb (*A*; *n* = 542), hindlimb (*B*; *n* = 55), head (*C*; *n* = 159), and trunk (*D*; *n* = 61). Each of the 4 body regions was further subdivided into 2 parts as follows: the hand and arm for the forelimb, the foot and leg for the hindlimb, the mouth and face/scalp for the head, and the dorsal and ventral sides for the trunk. The convergence RF is defined as the RF covering 2 subregions of each body region, for example, the “hand & arm” of forelimb neurons. Each of the 4-group neurons was subdivided into 3 groups: 2 with single RFs and 1 with convergence RFs (in the case of the forelimb neurons, they were divided into “hand,” “arm,” and hand & arm). No significant difference was found in the percentages of bilateral RFs (χ^2^-test, *P* > 0.05). Percentages (%) inside the black squares, percent of bilateral RFs.

As mentioned above, two types of RF enlargement were observed in the combined-type neurons. To confirm that the same phenomenon can be found generally in SII neurons, the following analysis was applied to neurons of the single-region type. We investigated the bilateral RF percentages among neurons of the single-region type when the neurons were classified into the convergence- and single-RF type (Fig. 5). For example, the forelimb was divided into two subregions—the hand and arm—and neurons with RFs confined to a single subregion (i.e., hand, arm) were defined as the single-RF type (“hand” and “arm”) and those with RFs covering both parts (“hand & arm”) were defined as the convergence-RF type. The forelimb neurons (*n* = 542) were divided into hand (*n* = 364), arm (*n* = 112), and hand & arm (*n* = 66). There were no significant differences in the numbers of neurons with bilateral RFs among the three subgroups (χ^2^-test, *P* > 0.05). The same analysis was applied to other body-region groups, and no significant difference was found between the neurons with convergence RFs and those with single RFs. Accordingly, we were unable to show that RF convergence is accompanied by an increased percentage of bilateral RFs in the single-region type, as observed for the combined-type neurons.

#### RF properties of the combined-type neurons.

In the following sections, we focused on describing the RF properties of the combined-type neurons of which RFs encompassed multiple body regions. Their most striking feature was the dominance of bilateral RFs; 253 out of the 282 combined-type neurons had RFs covering both sides of the body. As shown in Fig. 3, most bilateral RFs were symmetrically composed, with only one exception in this figure (No. 27). In this exception, the RF in the forelimb was confined to the contralateral side of the body, whereas the RFs in the digits of the hands and feet, as well as the mouth, were symmetrical. We compared the shapes of the bilateral RFs between both sides of the body for all of the bilateral RFs of the combined type. Because there were many neurons whose RF extent was not determined precisely, comparison of RFs between the two sides was done only at the subregional level defined in Fig. 1. For example, neurons with RFs in both sides of the hand were categorized as having a symmetrical RF of the hand, even when the digits that were included were not identified. As a result, we found only 10 asymmetrical RFs (4%) among 253 neurons. Bilateral RFs of the combined type were usually symmetrical, at least at the level of the body subregion defined in this study.

**Fig. 6.**
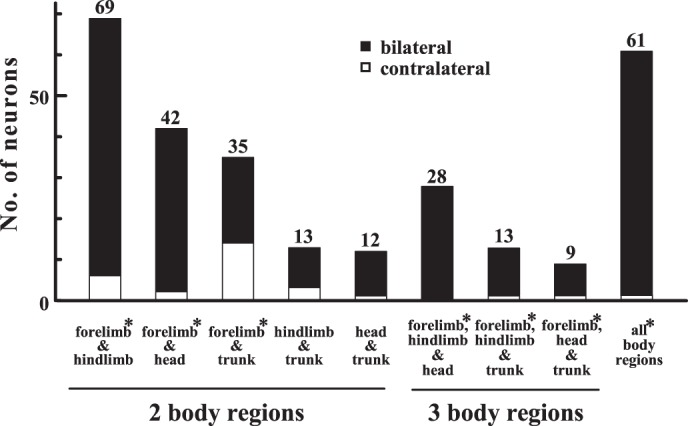
Classification of the combined-type neurons. The combined-type neurons were divided into 9 groups, according to the differences in the combinations of body regions. *Receptive field includes the forelimb.

The combined-type neurons were classified into nine groups, according to their RFs, with regard to the combination patterns of the four body regions (Fig. 6); the most numerous were RFs combining the forelimb and hindlimb (*n* = 69), followed by RFs covering all of the four body regions (*n* = 61). RFs of eight groups (shown in Fig. 6), among the nine groups of the combined type, included the forelimb, suggesting that the convergence of RFs among the four body regions usually occurs between the forelimb and other body regions. To confirm this, we calculated the ratio of the appearance of each of the four body regions. As a result, a total of 257 (91%) neurons among 282 had RFs, including the forelimb. In contrast, the percentages of other body regions were much lower: 65% for the hindlimb (*n* = 184), 50% for the head (*n* = 140), and 51% for the trunk (*n* = 143), respectively.

#### Limb type and trunk type in the combined-type neurons.

We further classified neurons of the combined type into two groups according to whether the RFs covered the trunk, that is, the *limb type* (without the trunk RF) and the *trunk type*. There were 139 *limb-type* neurons from three groups in Fig. 6 and 143 *trunk-type* neurons from six groups. These two types were quite different in terms of the percentages of skin and deep submodality neurons. There was a higher proportion of *limb-type* neurons receiving deep inputs (*n* = 73; 53%) compared with cutaneous inputs (*n* = 49; 35%). In contrast, more than two-thirds of the *trunk-type* neurons were skin neurons (*n* = 104; 73%), and the percentage of deep neurons was only 22% (*n* = 31). In the following sections, we present findings from further investigations of the RF properties of the *limb* and *trunk types*.

#### RFs of the limb-type neurons in the combined-type neurons.

Among the 139 *limb-type* neurons, 94% had bilateral RFs (*n* = 131). Approximately one-half of the 139 neurons was those of the “forelimb & hindlimb” type (*n* = 69; 50%), followed by the “forelimb & head” type (*n* = 42; 30%) and the “forelimb, hindlimb & head” type (*n* = 28; 20%). All of the RFs included the forelimb, and 70% included the hindlimb. As shown in Fig. 3, the RFs of the *limb type* (Nos. 14, 22, 24, 26–29, 31, and 33–35) were always composed of separated, small RFs on different body regions; we rarely found RFs covering almost the entire limbs, such as neurons 31 and 34 in Fig. 3. With regard to RFs on the limbs, they usually covered the distal parts, such as the hand and foot, and were mostly confined to the distal portions; these included neurons 22, 28, 29, 33, and 35 (Fig. 3). We found only seven neurons (5%) whose RFs on the limbs did not cover the distal portions.

**Fig. 7.**
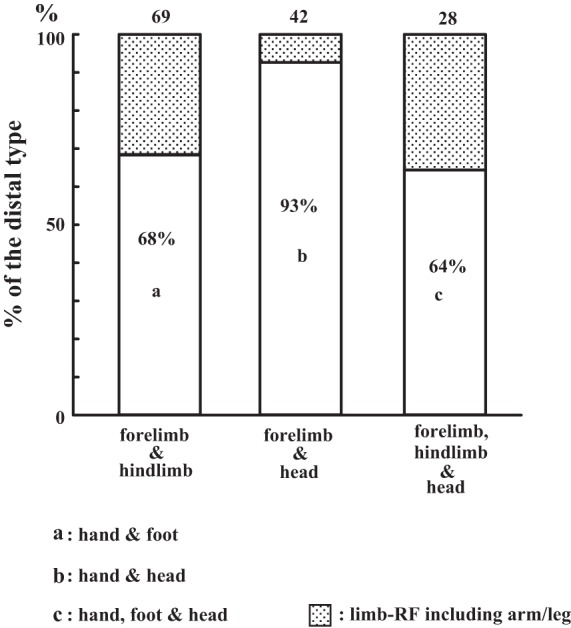
Percentages of the *limb-type* neurons whose receptive fields (RFs) are confined to the distal limb (distal type). Each of the 3 groups of *limb-type* neurons is divided according to whether their RFs on the limbs are confined to the hand/foot. “Hand & foot,” “hand & head,” “hand, foot & head,” neurons with RFs on the limbs confined to the hand/foot; “limb-RF including arm/leg” (shaded sections), neurons whose RFs on the limbs included the proximal parts of the limb (arm/leg).

We classified the *limb-type* neurons by whether or not the RFs on the limbs were restricted to the hand and foot (distal type). The percentages of the distal type in each group are shown in Fig. 7. Among the neurons of the forelimb & head (*n* = 42), the percentage of the distal type was 93% (*n* = 39), followed by 68% (*n* = 47) of the forelimb & hindlimb and 64% (*n* = 18) in the forelimb, hindlimb & head group. In total, 104 distal-type neurons (75%) were found among the 139 *limb-type* neurons.

RFs on the head of the distal type were usually restricted to the mouth regions (perioral and/or intraoral). Examples were neurons 22, 28, and 35 (Fig. 3). Among the distal-type neurons whose RFs covered the head (Fig. 7; *n* = 57), neurons with head RFs restricted to the mouth occupied 79% (*n* = 45). Accordingly, the combination of the different body portions in the *limb-type* neurons does not seem to occur randomly. There is a tendency for the distal portions of the limbs (the hand and foot) and the mouth to be combined with each other in the RFs.

#### RFs of the trunk-type neurons in the combined-type neurons.

**Fig. 8.**
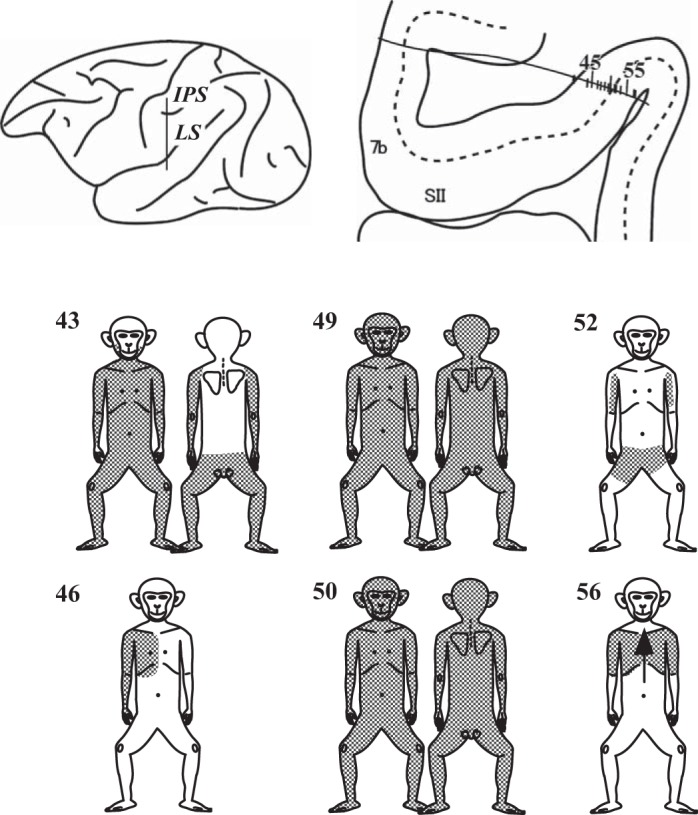
Examples of receptive fields (RFs) of the *trunk-type* neurons. Along the penetration depicted in the histological section, 6 out of 14 neurons are *trunk-type* neurons. Abbreviations and symbols are the same as those in Fig. 3. The trunk RF of No. 43 covers the ventral side of the neck and extends to the lower part of the head, i.e., the skin surface of the lower jaw. The hindlimb RF of No. 52 extends to a lower part of the belly.

Among the 143 *trunk-type* neurons, 85% (*n* = 122) had bilateral RFs, all of them crossing the midline of the trunk. As shown in Fig. 6, *trunk-type* RFs usually included the forelimb (*n* = 118; 83%); exceptions were the “hindlimb & trunk” (*n* = 13) and “head & trunk” (*n* = 12). Figure 8 shows examples of *trunk-type* RFs from one penetration in the same hemisphere shown in Fig. 3. In contrast to the *limb-type* RFs, RFs on different body regions of the *trunk type* were almost always interconnected with each other, as if the trunk RF expanded toward the other body regions continuously, such as in neurons 43, 46, 49, 50, and 56 in Fig. 8. We found only five exceptions (3%) whose RFs were composed of smaller, separated areas. An example is No. 52 in Fig. 8.

**Fig. 9.**
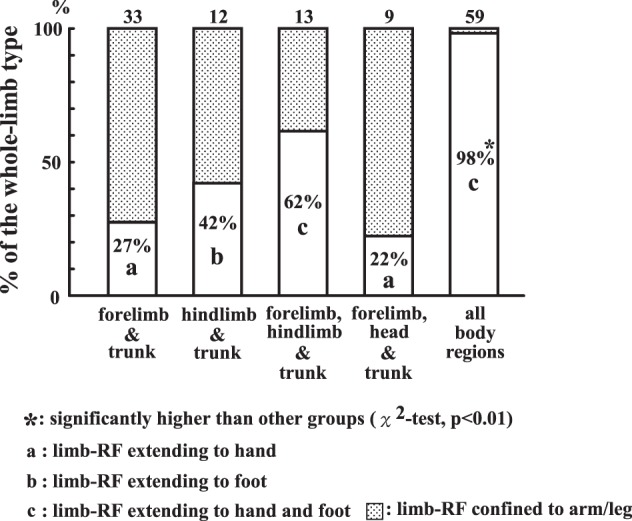
Percentages of the *trunk-type* neurons whose receptive fields (RFs) extend to the distal limb (whole *limb type*). Each of the 5 groups of *trunk-type* neurons is divided according to whether their RF extension reached the hand/foot. “Limb-RF extending to hand,” “limb-RF extending to foot,” and “limb-RF extending to hand and foot,” neurons with RFs on the limbs extending to the hand/foot. “limb-RF confined to arm/leg” (shaded sections), neurons whose RF extension did not reach the hand and foot.

Among the six groups of the *trunk type*, the most numerous were “all body-region” neurons (*n* = 61; 43%). Their RF extensions from the trunk to the limbs almost always reached both the hand and foot (Nos. 25 and 32 in Fig. 3 and Nos. 43, 49, and 50 in Fig. 8). This is quite different from other *trunk-type* neurons; that is, the RF extension did not reach the distal portion, as in neurons 52 and 56 in Fig. 8. Figure 9 shows the percentages of neurons whose RF extension reached the hand and/or foot (whole *limb type*). For this analysis, we excluded the 12 head & trunk neurons, because the RFs of these neurons lacked the limbs. Furthermore, RFs consisting of more than one separated part (*n* = 5), such as neuron No. 52 in Fig. 8, were also excluded. As a result, 126 neurons out of the 143 were analyzed. As shown in Fig. 9, the percentages of the whole *limb type* varied among the groups. The most striking one was an all body-region neuron whose RFs almost always extended to both the hand and foot (98%), with one exception, whereas the percentages varied between 22% and 62% in the other groups. These differences were significant (χ^2^-test, *P* < 0.01).

Neurons of all body regions almost always had bilateral RFs, as shown in Fig. 6. Thus those RFs seem to cover the entire body surface. However, we sometimes found exceptions, such as neuron No. 43 in Fig. 8, whose trunk RF did not cover the dorsal side, and the head RF covered only the skin surface of the lower jaw. In addition, there were some neurons whose head RFs were exclusive to the scalp region or whose limb RFs only covered the medial side of the limb.

#### Distribution of the combined-type neurons.

**Fig. 10.**
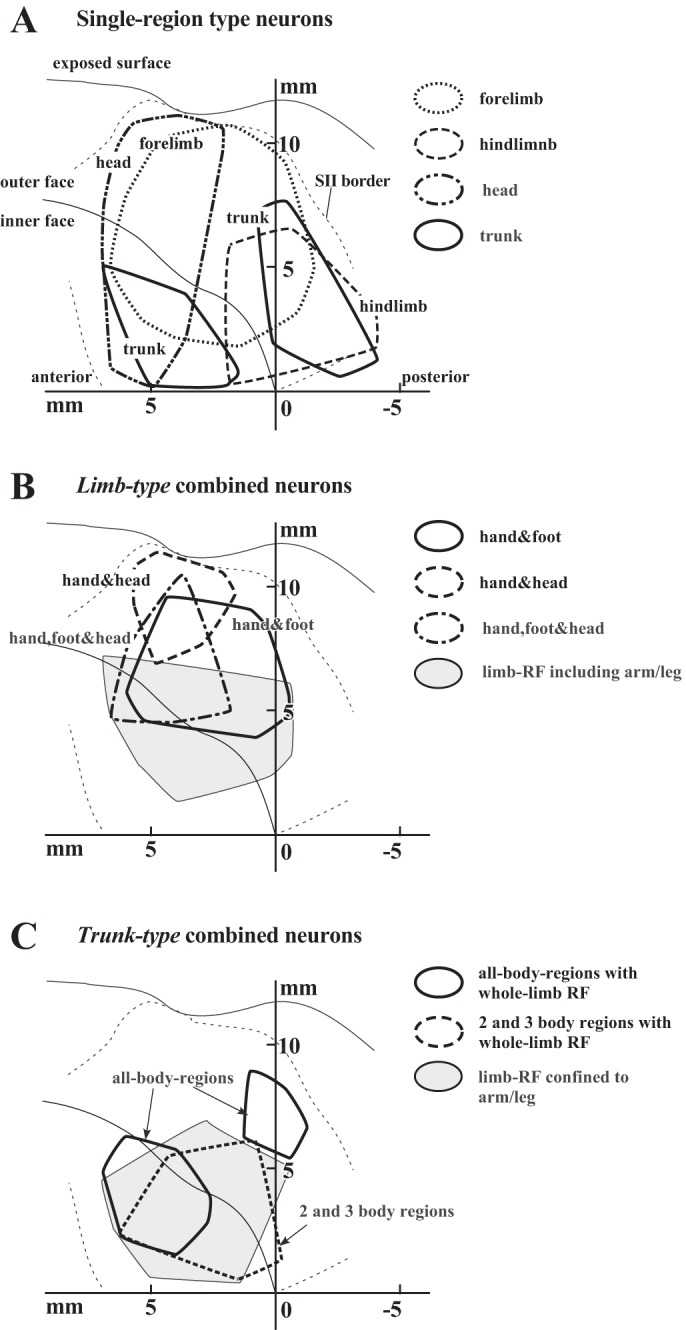
Distribution analyses of the combined-type neurons on the unfolded maps. Estimated recording sites on the coronal sections obtained from 6 hemispheres are plotted on the same unfolded maps (*n* = 799). Envelopes show the major distribution areas of neuron groups. *A*: the distributions of 4 neuron groups among the single-region type (*n* = 556), that is, the forelimb, hindlimb, head, and trunk, are shown. *B*: the distributions of the distal-type neurons (“hand & foot,” “hand & head,” and “hand, foot & head”) and the remaining neurons (“limb-RF including arm/leg”) among the *limb-type* neurons (*n* = 120) are shown. All of these categories are according to the classification in Fig. 7. *C*: the distributions of the whole *limb-type* neurons (“all-body-regions with whole-limb RF” and “2 and 3 body regions with whole-limb RF”) and the remaining neurons (“limb-RF confined to arm/leg”) among the *trunk-type* neurons (*n* = 123) are shown. All of these categories are according to the classification in Fig. 9. Other abbreviations and symbols are the same as those in Fig. 2.

Figure 10 shows the distribution of the combined-type neurons with the somatotopic map of SII based on the single region-type neurons. To make the distribution maps, estimated locations of neurons from six of the nine hemispheres (*n* = 799; see methods) were plotted onto the unfolded maps, and an envelope was drawn for each category to include 80% of the neurons to show the major distribution area of these neurons.

The somatotopic map in Fig. 10*A*, based on the single-region type (*n* = 556), is coincident with those previously reported: the head is represented in the rostral region and the hindlimb in the deeper part of the caudal region, with the large forelimb representation in the middle of SII (Burton et al. 1995; Fitzgerald et al. 2004, 2006a, b; Hihara et al. 2015; Krubitzer et al. 1995; Robinson and Burton 1980a; Taoka et al. 2013). The trunk representation occupies two separate areas: one in the deep area in the rostral half and the other relatively deep in the caudal-most region. Note the considerable overlaps among the different representations. An example of this overlapping representation is shown in Fig. 3. The first four neurons (Nos. 9–12) had RFs on the forelimb, followed by the head neuron (No. 13), but again, the forelimb neurons of the single-region type were recorded (Nos. 16–20). Because the focus of this study was on the combined-type neurons, we next describe the distribution of the *limb-type* and *trunk-type* neurons.

Figure 10*B* shows the distribution of the *limb-type* neurons (*n* = 120). As described earlier (Fig. 7), RFs on the limbs are usually restricted to the hand/foot (“distal type”) so that the distributions of the three groups of the distal type were analyzed on the unfolded map: hand & foot; hand & head; and hand, foot & head (Fig. 7). As shown in Fig. 10*B*, these three groups were mainly distributed in the outer face of the operculum, where the forelimb is represented (Fig. 10*A*). In addition, the distributions of neurons with RFs on the head (hand & head; hand, foot & head) were shifted to the rostral region corresponding to the overlapping region of the head and forelimb representations in Fig. 10*A*. On the other hand, the remaining *limb-type* neurons (RFs on the limbs not restricted to the hand/foot covering the proximal parts of the limb) were distributed around the border between the two faces (Fig. 10*B*), broadly along the rostrocaudal axis covering the deeper part of the forelimb representation in Fig. 10*A*, although those four envelopes overlapped with each other.

Figure 10*C* shows the distribution of the *trunk-type* neurons (*n* = 123). On this map, the *trunk-type* neurons were classified into two categories, according to whether the RF extensions reached the hand and/or foot (i.e., neurons of the whole *limb type* and neurons of “limb-RF confined to arm/leg” in Fig. 9). The whole *limb type* was further subdivided into the two categories: neurons of all body regions and the other neurons with RFs covering two or three body regions (both in Fig. 10*C*). As also shown in Fig. 10*C*, all body-region neurons are distributed in two restricted regions: one almost confined to the rostral part of the inner face and the other in the outer face near the caudal end of SII. These two areas occupy the rostral-most part of the two trunk representations in Fig. 10*A*. In contrast, the distributions of the other two groups, “2 and 3 body regions with whole-limb RF” and the remaining neurons with RFs on the limb confined to the arm/leg (Fig. 10*C*), highly overlapped.

## DISCUSSION

The present study showed the properties of neurons with large RFs in SII of Japanese macaque monkeys. Among the neurons whose RFs and submodality preferences were identified, most RFs (74%) were confined to a single-body region (single-region type) when the entire body was divided into four distinct body regions (forelimb, hindlimb, trunk, and head). However, 26% of the neurons had large RFs covering more than one body region (combined type). We investigated the RF properties of the combined type and found that there are two tendencies for generating large RFs by integrating somatic information from the different body regions: either *1*) the distal parts of the limbs (the hand and foot) and the mouth are connected with each other, or *2*) the trunk RFs extend toward the other body regions to cover the entire body surface. Finally, we showed that neurons with RFs, with the two tendencies, are distributed in specific subregions of SII, suggesting functional subdivisions of SII.

### 

#### Body categorization for the systematic analysis of large RFs in the present study.

Large RFs in SII of macaque monkeys have been reported previously, such as RFs covering the chest and upper limbs (Whitsel et al. 1969), the trunk and other body parts (Poranen and Hyvarinen 1982; Robinson and Burton 1980b), and the hand and face (Taoka et al. 2013). However, systematic analyses of the RF integration from different body regions and from both hemibodies (bilateral integration) have never been reported before. To evaluate quantitatively the extent of RF convergence, we classified the entire body into four body regions and grouped SII neurons according to whether RFs covered more than one body region (combined type) or were confined to one body region (single body-region type). The combined-type RFs are characterized by the dominance of bilateral RFs (90%). Thus we concluded that the convergence of RFs from different body regions was accompanied by the integration of somatic information from both hemibodies (bilateral convergence). However, this simultaneous occurrence of two different RF convergences was not observed among the neurons of the single body-region type (Fig. 5); that is, RF convergence from different subregions within the same body region (e.g., hand & arm in Fig. 5) was not accompanied by bilateral convergence. This suggests that different mechanisms of somatosensory information processing probably work for the RF enlargements in the single-region and combined types. This finding was enabled by the body categorization adopted in the present study, suggesting its validity for the systematic analysis of RF enlargement in SII.

#### Comparison of large RFs between SII and the postcentral somatosensory cortex.

Iwamura et al. (1993) reported an increased RF size and complexity along the rostrocaudal axis in the hand region of the postcentral somatosensory cortex and found RFs covering multiple digits in areas 2 and 5, as well as bilateral RFs (Iwamura et al. 1993, 1994). In addition, RFs on the proximal forelimb, face, or trunk were found in deeper parts of the anterior bank of the intraparietal sulcus. RFs combining different body parts have been reported in more medial postcentral regions representing the arm, trunk, and hindlimb (Duffy and Burchfield 1971; Leinonen et al. 1979; Sakata et al. 1973; Taoka et al. 1998, 2000). Taoka et al. (1998) found large RFs covering the trunk and forelimb in areas 2 and 5 in the arm/trunk regions. In the lateral-most part of the hindlimb region that neighbors the region representing the trunk, Taoka et al. (2000) found neurons with RFs both on the forelimb and hindlimb in areas 2 and 5. Furthermore, in the medial wall of the postcentral gyrus, neurons with RFs on the leg extending to the lower part of the trunk were also reported in areas 2 and 5 (Taoka et al. 2000). Although such RFs were more common among bilateral RF neurons than among contralateral RF neurons (Iwamura 1998; Iwamura et al. 2001a, 2002; Taoka et al. 1998, 2000), the incidence of such RFs was very low (7% at most; Taoka et al. 1998, 2000). Furthermore, their RFs usually covered only two body regions neighboring each other; that is, RFs covering both the forelimb and hindlimb were found only in the lateral-most part of the hindlimb region with low incidence (under 1%) (Taoka et al. 2000), and neurons with RFs covering three or four body regions have not been reported in the postcentral somatosensory cortex.

Although RF integration that generates large RFs is obvious in the hierarchical information processing in the postcentral somatosensory areas (Iwamura 1998; Iwamura et al. 2001a, 2002), the convergence of RFs is usually confined in a single-body region. Furthermore, neurons having one of the two tendencies of RF enlargement mentioned above have never been reported in the postcentral somatosensory area. With the consideration of the higher percentage of bilateral RFs (Iwamura et al. 2001a, 2002; Robinson and Burton 1980b; Whitsel et al. 1969), we conclude that RF enlargement in SII is more developed than in the postcentral somatosensory cortex in both encompassing multiple body regions and bilater al integration.

#### Implications of functional roles to integrate information from different body regions.

The role of SII in tactile-object recognition is generally accepted, and many neurophysiological studies using macaque monkeys have supported this idea [reviewed by Hsiao (2008)]. On the other hand, recent human imaging studies often demonstrated that SII activity is related to sensorimotor integration rather than object recognition (Binkofski et al. 1999; Hinkley et al. 2007; Wasaka et al. 2005, 2007), although monkey neurophysiological studies supporting this idea have rarely been reported. Taoka et al. (2013) found SII neurons that responded to both hand and mouth movements (“hand & mouth movement” neurons) around the area bordering the representations of the hand and face in the macaque SII. When the monkey performed a simple feeding task (retrieving and eating food from an experimenter's hand), hand & mouth movement neurons became active during both reaching/grasping and eating. More than one-half of the hand & mouth movement neurons had RFs in both the hand and face. Furthermore, ∼95% of these neurons showed bilateral activity during hand movements. Because the hand & mouth movement neurons rarely responded when objects were touched (experimenter's hand and food), these neurons do not seem to serve the function of detecting object features (Taoka et al. 2013). That study did not investigate the hindlimb to identify RFs and movement-related activity, but the neurons seem to have similar characteristics to the distal type of the *limb-type* neurons (i.e., hand & head; hand, foot & head) in the present study because of the dominance of deep neurons among the distal-type neurons and their distribution in the rostral area of the outer face where the representations of the head and the forelimb overlap (Fig. 10, *A* and *B*). These findings suggest that one of the functions of the *limb-type* neurons is to contribute to motor execution using the distal part of the limbs and mouth, which are the body parts for touching and manipulating objects in the external environment.

On the other hand, we could not explore the implications of the role of *trunk-type* neurons. One of the interesting features of the *trunk-type* neurons is that their bilateral RFs composing 85% of the *trunk type* always crossed the midline of the trunk. Representations of the trunk midline have been reported even in the early stage of the somatosensory information processing, that is, area 3b (Conti et al. 1986; Taoka et al. 1998); however, they are usually restricted to the close vicinity of the midline. In later-stage information processing (i.e., in areas 2 and 5), larger RFs on the bilateral trunk, including the midline, were frequently observed (Taoka et al. 1998). It has been suggested that these RFs crossing the midline contribute to the unified perception of the body region (Iwamura 2000; Iwamura et al. 2001a, 2002; Manzoni et al. 1989) whose left and right sides are separately represented in SI. This RF enlargement crossing the midline progresses further in SII and finally results in the whole-body representation.

Large RFs covering the four body regions have been reported in some macaque neurophysiological studies in other cortical areas (Duhamel et al. 1998; Hikosaka et al. 1988; Leinonen et al. 1979; Robinson and Burton 1980a); however, systematic analyses of the response properties of the neurons of all body regions were not performed in these studies. The present study is the first to describe the detailed features of all body-region neurons and their distribution in the nonhuman primate cortex.

The whole-body representation is often discussed from the perception of one's body as a unified entity that requires the integration of somatic and visual information from the entire body (Blanke 2012; Ehrsson 2012). A recent macaque neurophysiological study performed by Hihara et al. (2015) found that visually responsive neurons are distributed widely in SII. Until now, however, we failed to find bimodal neurons among the all body-region neurons. It is also possible that information of the somatic whole-body representation in SII is conveyed to other cortical areas related to the whole-body perception. For example, SII is connected to the ventral premotor area (Cipolloni and Pandya 1999; Disbrow et al. 2003; Matelli et al. 1986), where the full-body representation with visuosomatic bimodal activities has been reported in human brain-imaging studies (Gentile et al. 2015; Petkova et al. 2011). However, macaque studies in the premotor area have not demonstrated the somatic whole-body representation. At present, we do not have any neurophysiological results supporting the functional role of the all body-region neurons. However, the dominance of all body-region neurons among the *trunk type* and the high percentage of skin neurons and their restricted distribution suggest a novel role for all body-region neurons.

## GRANTS

Support for this study was provided by Grants-in-Aid for Scientific Research from the Ministry of Education, Culture, Sports, Science and Technology, Japan (Nos. 08279101 and 17500205).

## DISCLOSURES

No conflicts of interest, financial or otherwise, are declared by the authors.

## AUTHOR CONTRIBUTIONS

M. Taoka, T.T., S.H., M. Tanaka, A.I., and Y.I. conception and design of research; M. Taoka, T.T., S.H., M. Tanaka, A.I., and Y.I. performed experiments; M. Taoka, T.T., S.H., M. Tanaka, and A.I. analyzed data; M. Taoka, T.T., S.H., M. Tanaka, A.I., and Y.I. interpreted results of experiments; M. Taoka, T.T., and M. Tanaka prepared figures; M. Taoka drafted manuscript; M. Taoka, T.T., S.H., M. Tanaka, A.I., and Y.I. edited and revised manuscript; M. Taoka, T.T., S.H., M. Tanaka, A.I., and Y.I. approved final version of manuscript.
